# Survey of the Parasite *Toxoplasma gondii* in Human Consumed Ovine Meat in Tunis City

**DOI:** 10.1371/journal.pone.0085044

**Published:** 2014-01-10

**Authors:** Sonia Boughattas, Khaled Ayari, Tongmin Sa, Karim Aoun, Aida Bouratbine

**Affiliations:** 1 Department of Environmental and Biological Chemistry, College of Agriculture, Life and Environmental Sciences, Chungbuk National University, Cheongju, Chungbuk, South Korea (Republic of); 2 Parasitology Laboratory, Institut Pasteur Tunis, Tunis, Tunisia; Albert Einstein College of Medicine, United States of America

## Abstract

Toxoplasmosis has been recognized as parasitic zoonosis with the highest human incidence. The human infection by the parasite can lead to severe clinical manifestations in congenital toxoplasmosis and immunocompromised patients. Contamination occurs mainly by foodborne ways especially consumption of raw or undercooked meat. In contrast to other foodborne infections, toxoplasmosis is a chronic infection which would make its economic and social impact much higher than even previously anticipated. Ovine meat was advanced as a major risk factor, so we investigated its parasite survey, under natural conditions. Serological MAT technique and touchdown PCR approaches were used for prevalence determination of the parasite in slaughtered sheep intended to human consumption in Tunis City. The genotyping was carried by SNPs analysis of SAG3 marker. Anti-*Toxoplasma* antibodies were present in 38.2% of young sheep and in 73.6% of adult sheep. Molecular detection revealed the contamination of 50% of ewes’ tissue. Sequencing and SNPs analysis enabled unambiguous typing of meat isolates and revealed the presence of mixed strains as those previously identified from clinical samples in the same area. Our findings conclude that slaughtered sheep are highly infected, suggesting them as a major risk factor of *Toxoplasma gondii* transmission by meat consumption. Special aware should target consequently this factor when recommendations have to be established by the health care commanders.

## Introduction

*Toxoplasma gondii (T.gondii)* is a cosmopolite parasitic protozoon. It infects all warm-blooded vertebrate species, including humans. Human infection can leads to drastic consequences during congenital transmission [1] and in immunocompromised patients [2]. Transmission occurs mainly by foodborne way through the ingestion of contaminated vegetable /water with oocysts, as well as the ingestion of contaminated raw / undercooked meat with tissue cysts [3]. The European Food Safety Authority (EFSA) has recognized toxoplasmosis as parasitic zoonosis with the highest human incidence [4]. Researchers considered the consumption of undercooked infected meat as the biggest risk and suspected ovine meat, to be a major risk factor for human infection [5]. In our country, Tunisia (North of Africa), data still fragmentary about the survey of this parasite in slaughtered ovine. No previous study investigated relevantly the detection the parasite in the meat and its genotyping for epidemiological correlation with clinical data [6]. This is what we propose to cover by our present work to check if ovine meat could or not be a risk factor for human contamination in Tunis City, the capital of Tunisia.

## Materials and Methods

### Sheep sera

Blood samples were collected, in slaughterhouse upon the individual owners request, from 217 lambs (3–11 months old) and 125 ewes (≥ 1-year-old) intending for human consumption in the neighborhood of Tunis City. No endangered or protected species have been included in our study. The blood was drawn via the jugular vein from each animal at the time of slaughter by exsanguinations as approved by the Tunisian National Ethics Committee. Sera were separated after centrifugation 2300 x *g* for 10 min and stored at –20°C until analysis.

Serum samples were initially screened at 1∶20 and 1∶200 dilutions with the modified agglutination test (MAT) using whole formalin-preserved tachyzoites as antigen [7]. Seropositive sera were then end-titrated using 2-fold dilutions.

### Sheep tissues

We purchased 72 ewes heart from different butchers in Tunis City. Hearts were collected in individual plastic bags and kept refrigerated (+4°C). Intra-cardiac blood or meat juices were collected for serological examination by MAT technique. When the serological sampling wasn't possible, tissue specimen were grouped in pools which will be, like seropositive hearts, subjected to enzymatic treatment for the molecular identification.

Briefly, heart tissue was ground in 2 volumes (w/v) of 0.9% NaCl and digested 2:3 (v/v) with 2.5% fresh trypsin solution in PBS. Lysis was achieved after incubation during 1h30/150 rpm at 37°C. The product was then filtrated twice, pelted by cold centrifugation 10 min at 2500 rpm, washed and resuspended in saline containing Penicillin G/Streptomycin.

### DNA extraction

700 µl of the treated pellet was subjected to DNA extraction protocol. The same volume of aqueous lysis buffer (100 mM Tris HCl pH 8, 1.4 M NaCl, 20 mM EDTA pH8, 2% CTAB) containing fresh 70 µl of Proteinase K (10 mg/ml) was added for overnight incubation in 65°C water bath. Lysate extraction was done by chloroform (v/v). After the cold centrifugation at maximum speed, clear aqueous phase must be recovered otherwise chloroform treatment should be repeated for the upper phase. DNA was precipitated by the addition of Sodium acetate at a final concentration of 0.3 M and 0.6 volume of isopropanol, followed by 15 min incubation at –80°C. After cold centrifugation, wash step by cold 70% ethanol, the pellet was dried and resuspended in 70 µl warm TE buffer.

### PCR reaction

*T.gondii* diagnostic was carried out by B1 PCR using the primers B22: AACGGGCGAGTAGCACCTGAGGAGA and B23: TGGGTCTACGTCGATGGCATGACAAC. DNA extract was tested in duplicate with the use of an internal amplification control (0.1pg of reference DNA) to enable the identification of false negative PCR results. Amplification was performed in a final volume of 25 µl with 10X PCR buffer, 2.5 mM MgCl2, 200 µM dNTP each, 0.6 mg BSA, 10 pmol of primers, 1U of Gold Taq Polymerase (Applied Biosystems) and 5 µl of sample DNA.

Amplification program started with preliminary steps of 2min at 50°C then of 6min at 95°C and followed by 40 cycles of 30sec at 94°C, 30sec at 57°C and 1min at 72°C adding 1sec/cycle. The final elongation step was achieved after 7min at 72°C. Reaction products were resolved on 3% agarose gel.

### Genotyping

Single nucleotide polymorphism (SNPs) was investigated for the positive tissues at the marker SAG3 as described elsewhere [8]. Sample profiles were compared to profiles of references strains RH, PRU and NED kindly provides by Toxoplasma BRC (Limoges-France). Phylogenetic network analysis was performed using the software SplitsTree 4 [9].

### Statistical test

The difference in the seroprevalence between the age categories was analyzed by the chi-square test [10].

## Results

Antibodies to *T. gondii* (Table 1) were found in 83 of 217 lambs (38.2%) in titers of 20 (25 lambs), 40 (23 lambs), 80 (12 lambs), 160 (9 lambs), 320 (3 lambs) and ≥ 640 (11 lambs). In ewes, positives sera were 92 of 125 (73.6%) with 56 samples within 1∶20 dilution, 11 within 1∶40, 3 within 1∶80, within 1∶160, one sample within 1∶320 and 11 within ≥1∶640 dilution. Statistical analysis showed that difference in prevalence observed between ages was significant (p <0.001).

**Table:** Table 1. Summary of antibodies prevalence.

	Age	Ni	N+	N-	%
Lambs	3–11 months	217	83	134	38.2
Ewes	≥ 1-year-old	125	92	33	73.6

Ni: Initial number of tested serums; N+: Number of sera with positive reaction; N–: Number of sera with negative reaction; %: percentage of seroprevalence.

In an attempt to identify *T.gondii* from sheep tissue, we weren't able to do serological sampling for 18 hearts; consequently tissues were grouped in 3 pools: ShrTgTnP1, ShrTgTnP2 and ShrTgTnP3. For the remaining 54 tissues, we detected *Toxoplasma* antibodies in 34 (63%) hearts from ewes slaughtered in Tunis City within dilutions 1∶20 in 5 samples, 1∶40 in 5 samples, 1∶80 in 6 samples, 1∶160 in 14 samples, 1∶320 in 3 samples and titter ≥ 1∶640 in 1 ewe.

**Figure 1:**
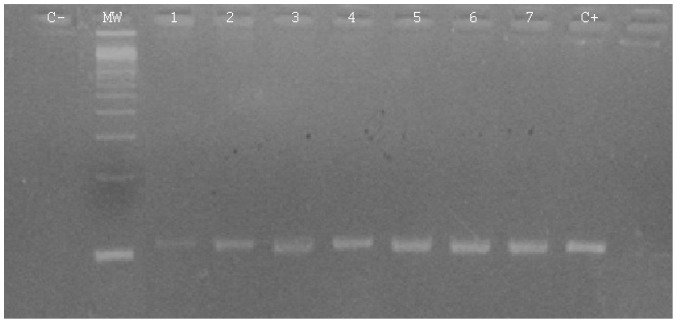
Electrophoretic pattern of the direct PCR on sheep hearts. C- corresponds to negative control of PCR; MW: 100bp ladder; 1–9: amplification of different sheep isolates and C+ corresponds to the positive control of the reaction.

With the new extraction protocol, inhibition reaction wasn’t observed, however best visualization of the result was observed after optimization of DNA dilution [data not shown]. Parasite DNA amplification was obtained in 17 hearts (50%) and in 1 of the 3 tissue pools by visualizing the 114 bp band after agarose gel electrophoresis (Figure 1). *T.gondii* was detected mainly from tissues with high antibodies titer (Table 2).

**Table:** Table 2. Data about positive sheep heart samples.

Strains	MAT titer	SAG3 type
ShrTgTn P1	ND	II/III
ShrTgTn 1	160	III
ShrTgTn 2	40	I
ShrTgTn 8	160	II
ShrTgTn 10	320	III
ShrTgTn 17	20	III
ShrTgTn 20	40	II/III
ShrTgTn 24	160	II/III
ShrTgTn 30	160	III
ShrTgTn 31	160	II/III
ShrTgTn 34	160	III
ShrTgTn 35	160	III
ShrTgTn 37	320	III
ShrTgTn 42	20	III
ShrTgTn 44	80	II
ShrTgTn 49	160	III
ShrTgTn 53	40	III
ShrTgTn 54	20	I

ND: not done.

**Figure 2:**
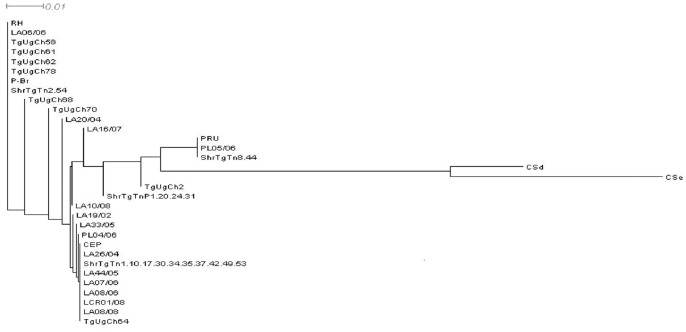
SplitsTree network of toxoplasmic strains at the marker SAG3. RH, PRU and CEP represent reference strains of type I, II and III respectively. TgUgCh: strains isolated form free-ranging chicken in Uganda. LA, PL, LCR: Tunisian clinical strains. ShrTgTn: strains isolated from sheep in Tunisia. CSe and CSd: atypical strains.

Amplification was retained for all the 18 samples by SAG3 marker. The 225bp products were purified and subjected to NciI restriction reaction. Comparison between samples and reference strains profile’s showed: profiles identical to strain I profile within two samples, two other samples were typed as strain II and ten samples revealed genotype III. The remaining 4 samples, advanced complex profiles suggesting the presence of type II and III simultaneously (Table 2). Mixed infection was confirmed by sequencing (Table 3) and well demonstrated by the intermediate position of isolates within the phylogenetic network (Figure 2). Sequences analysis revealed also homology with clinical isolates identified in our previous work with the clustering of Tunisian clinical and environmental samples in the phylogenetic network (Figure 2).

**Table 3:** Summary of strains polymorphism at SAG3 marker.

	SNPs polymorphism positions at the marker SAG3								
Strains/Positions	64	84	88	120	127	129	136	144	159
RH Type I	C	G	A	T	G	G	A	G	A
PRU Type II	T	A	G	T	A	G	C	C	G
NED Type III	C	G	A	C	G	A	C	C	A
ShrTgTn 24	Y	R	R	Y	R	R	C	C	R

## Discussion

Previous studies showed that selected areas for which seroprevalence was high in sheep corresponded to those for which seroprevalence was also high in humans [11]. The direct link between sheep infection and human contamination at the area level is difficult to assess because meat is not always consumed in the same area where animals are slaughtered. Nevertheless, the high seroprevalence of the parasite in several species within the same areas could be related to an intense circulation of the parasite, perhaps due to specific environmental conditions (felids densities, climatic specificities, etc.). Those areas may be considered as “risky areas” for toxoplasmosis infection and specific investigations should be conducted [12]. In Tunisia, Tunis City (located in the north), is the largest city and the densest human agglomeration. Within it, *Toxoplasma* prevalence was estimated to 47.7% among pregnant women [13]. Regarding prevalence in sheep, two published studies are reported. The first work published in 1970 [14] advanced 75% dye-test sero-prevalence in slaughtered sheep, regardless animal age. Recently, another analysis investigated the prevalence of the parasite within age, sex and locality differentiation [15]. Low prevalences were reported in northern cities: 1.8% by ELISA and 12.7% by nPCR, with insignificant difference between young and old animals. Consequently our present work is, to our knowledge, the first survey in Africa of *T.gondii* prevalence in ovine livestock using combined serological analysis, molecular detection and genotypic characterization.

For the serological test, we opted for MAT technique as its relative sensitivity is the highest when performed on cardiac fluids [16]. Contradictory to what was published recently in our country, seroprevalence among adult sheep was significantly higher than among young sheep. This confirms previous reports indicating a higher risk of exposure as age increases in animals [17], [18] and humans. The seroprevalence in lambs cannot be related to a transfer of maternal antibodies through colostrums, which occurs within 3 months after birth [19]. Risk factors for infection with *T.gondii* in sheep are within the age of 7 months or older, suggesting that most of the animals acquired the infection progressively after birth [12].

In our study, the advanced prevalence of antibodies in Tunisian ovine was higher to those observed as average in African countries: 5.6% in South Africa [20], 6.7% in Nigeria [21], 22.9% in Ethiopia [22] and 32.2% in Ghana [23]. Moreover, unexpectedly seroprevalence in Morocco, a close country, is about 27.6% [24] and 43.7% in Egypt [25], other geographically close country. However similar results are observed in some localities from: Turkey 66.66% [26], France 65.5% [27], Switzerland 61.6% [28] and from Suriname 67% [29]. The studied areas are known to be warm, moist tending to favour the survival of oocysts. Indeed, Tunis City is located in humid zone with an average annual rainfall over 400 mm. Its climatic characteristics increase the chance of viability of the parasite and as result, confer generally high prevalence. In the present study, the fact that 16.8% of the tested sheep had a high antibodies titer (≥160) is an indication of frequent exposure to the parasite in farms.

To explore whether this high prevalence could be a risk factor or not to human contamination, we investigated to study the prevalence of the parasite in the ovine meat. The most sensitive tissues of the sheep to *T.gondii* are the brain and the heart. Since adult-sheep hearts are more frequently consumed in Tunisia, we focused our study on the analysis of ewes hearts purchased from butchers so directly intended to human consumption. We tested antibodies in meat juice since it was described as: reasonable alternative to serum when testing carcasses and relevant matrix for toxoplasmosis survey in meat [30], [16]. The serological result was in concordance with seroprevalence study showing high rate of anti-*T.gondii* antibodies in adult animals. The positive tissues were digested by trypsin to liberate the eventual toxoplasmic cysts present in the meat. We had chosen to use the CTAB in the lysis step of our DNA extraction protocol. This reagent, frequently used in plant extraction protocols [31], is described with high power of binding and purifying DNA from other tissues components. Even without the use of phenol which lead predict to higher protein contamination, the efficiency of CTAB was well demonstrated by obtaining a clear upper phase well separated from the proteins ring and the organic phase. The extracted DNA was enough pure to not induce any inhibition during PCR diagnosis reaction.

For the parasite detection we used the primers B22/B23 as they were described the most sensitive ones even in tissues [32]. *T.gondii* was detected in the half of the seropositives tissues which indicate the high rate of meat contamination in contrast to what was reported previously in our country [15]. The distribution of *T.gondii* parasites within same tissue is random, and parasite density may be low. Therefore, a negative result has to be interpreted carefully because it is possible that the parasite could be present in unexamined parts of the target tissue. The Gharbi *et al* team analysed the apex of the heart for *Toxoplasma* detection, however our approach investigated the detection in the whole animal heart which can explain the higher prevalence that we obtained. The same rate was also observed in Netherland where molecular detection advanced the contamination of 50% of seropositive hearts [33]. However the used technique was combination of magnetic capture and PCR, much more complex than the touchdown PCR we used.

All the positive samples were able to be amplified by the marker SAG3 for genotyping [34]. SAG3 is one of the most sensitive described markers with detection level up to 5 parasites /sample. It is also a frequently used RFLP marker in the literature, offering thus abundant data about worldwide *T.gondii* genotyped isolates. The observed genotypes within our meat isolates concord with previous ones identified from human clinical cases in the same area [6]. The presence of mixed genotypes could be explained by the presence of a pool of tissues in one case, but its presence in three single samples underlines more that mixed infections are frequents on African content [35], [6], [1]. The phylogentic analyses demonstrate well the close relation between our isolates and African ones (TgUgCH) as it demonstrates the clustering between Tunisian clinical and sheep isolates.

## Conclusions

According to Tunisian traditions, pregnant women are encouraged to consume undercooked meat (ovine, free ranging chicken and horse meat) to have “healthier baby”. In previous work, we investigated the prevalence of the parasite among horses; however the low observed prevalence disqualify it from risk factors list [36]. Within our current study, considering the presence of *T.gondii* in slaughtered sheep and the clustering of the identified strains with those described from Tunisian clinical samples, ovine meat is suggesting as a major risk factor of human contamination in Tunis City. Special aware must be taken, especially with the identification of mixed genotypes which are presumed to be more virulent than clonal types I, II and III [1].
